# Leptin-Induced Endothelium-Dependent Vasorelaxation of Peripheral Arteries in Lean and Obese Rats: Role of Nitric Oxide and Hydrogen Sulfide

**DOI:** 10.1371/journal.pone.0086744

**Published:** 2014-01-24

**Authors:** Anna Jamroz-Wiśniewska, Arieh Gertler, Gili Solomon, Mark E. Wood, Matthew Whiteman, Jerzy Bełtowski

**Affiliations:** 1 Department of Pathophysiology, Medical University, Lublin, Poland; 2 Department of Neurology, Medical University, Lublin, Poland; 3 Institute of Biochemistry, Food Science and Nutrition, The Hebrew University of Jerusalem, Rehovot, Israel; 4 Biosciences, College of Life and Environmental Sciences, University of Exeter, Exeter, United Kingdom; 5 University of Exeter Medical School, St Luke’s Campus, Exeter, Devon, United Kingdom; Medical School of Hannover, Germany

## Abstract

Adipose tissue hormone leptin induces endothelium-dependent vasorelaxation mediated by nitric oxide (NO) and endothelium-derived hyperpolarizing factors (EDHF). Previously it has been demonstrated that in short-term obesity the NO-dependent and the EDHF-dependent components of vascular effect of leptin are impaired and up-regulated, respectively. Herein we examined the mechanism of the EDHF-dependent vasodilatory effect of leptin and tested the hypothesis that alterations of acute vascular effects of leptin in obesity are accounted for by chronic hyperleptinemia. The study was performed in 5 groups of rats: (1) control, (2) treated with exogenous leptin for 1 week to induce hyperleptinemia, (3) obese, fed highly-palatable diet for 4 weeks, (4) obese treated with pegylated superactive rat leptin receptor antagonist (PEG-SRLA) for 1 week, (5) fed standard chow and treated with PEG-SRLA. Acute effect of leptin on isometric tension of mesenteric artery segments was measured *ex vivo*. Leptin relaxed phenylephrine-preconstricted vascular segments in NO- and EDHF-dependent manner. The NO-dependent component was impaired and the EDHF-dependent component was increased in the leptin-treated and obese groups and in the latter group both these effects were abolished by PEG-SRLA. The EDHF-dependent vasodilatory effect of leptin was blocked by either the inhibitor of cystathionine γ-lyase, propargylglycine, or a hydrogen sulfide (H_2_S) scavenger, bismuth (III) subsalicylate. The results indicate that NO deficiency is compensated by the up-regulation of EDHF in obese rats and both effects are accounted for by chronic hyperleptinemia. The EDHF-dependent component of leptin-induced vasorelaxation is mediated, at least partially, by H_2_S.

## Introduction

Increased adiposity and obesity are the leading causes of arterial hypertension and atherosclerosis, the most prevalent cardiovascular diseases associated with endothelial dysfunction [1], [2]. Several studies in the last couple of decades have clearly shown that abnormal production of adipose tissue hormones (adipokines) plays an important role in obesity-associated abnormalities of vascular function (reviewed in [3]–[5]). Leptin, the first and most highly characterized adipokine, not only inhibits food intake and stimulates energy expenditure but also affects many other physiological processes including reproduction, inflammatory and immune reaction, bone metabolism and vascular homeostasis [6]–[9]. However, the precise effect of leptin on vascular tone is controversial. Acutely administered leptin has been shown to induce endothelium-dependent and endothelium-independent vasorelaxation [10], [11], but at higher concentrations and acting over a long time it also impairs vasodilation induced by other agonists such as acetylcholine [12], [13]. In general, under normal physiological conditions leptin has no acute effect on blood pressure because it activates both pressor (sympathetic nervous system, SNS) and depressor (vasodilation and natriuresis) mechanisms in the balanced manner. In contrast, conditions associated with chronic hyperleptinemia such as obesity, type 2 diabetes, chronic kidney disease, preeclampsia and obstructive sleep apnea are characterized by endothelial dysfunction and/or hypertension [14]–[16]. However, it is unclear if the enhancement of detrimental effects of leptin such as SNS and oxidative stress or reduced leptin-induced vasorelaxation due to leptin resistance is more important in the pathogenesis of vascular dysfunction.

We have previously demonstrated that chronic hyperleptinemia either induced in lean rats by exogenous administration of the hormone or “endogenous” hyperleptinemia associated with obesity induced by highly palatable diet impairs the acute vascular NO-mimetic effect of leptin [17], [18]. Nevertheless, in the early stage of obesity when insulin sensitivity is not impaired, this is compensated by the up-regulation of endothelium-derived hyperpolarizing factor (EDHF)-mediated vasorelaxation [18]. In later phase of obesity when insulin sensitivity is compromised, both NO- and EDHF-mimetic effects of leptin are impaired leading to unopposed stimulation of SNS and blood pressure elevation [18]. These conclusions were based primarily on blood pressure measurement after systemic administration of leptin and respective inhibitors, and/or measurement of NO metabolites and cGMP in the aortic wall. In addition, the mechanisms of leptin-induced EDHF were not clarified. With the above observations in mind, in the present study we examined the involvement of NO and EDHF components in leptin-induced vasorelaxation by directly measuring vascular tone ex vivo. Because NO is progressively replaced by EDHF while moving from large conduit to small resistance arteries [19], we used mesenteric artery rings since they are expected to exhibit both these mechanisms. In addition, we investigated whether the impairment of acute NO-mimetic effect of leptin in obesity was accounted for by chronic hyperleptinemia. For this purpose, we developed mono-pegylated super active rat leptin antagonist (D23L/L39A/D40A/F41A mutant) and treated a subgroup of obese and normally-fed rats to switch-off the endogenous leptin signaling. Finally, we examined the mechanism of NO-independent leptin-induced vasorelaxation and demonstrated that leptin-induced EDHF is mediated by endogenous hydrogen sulfide (H_2_S).

## Materials and Methods

### Preparation and Characterization of Non-pegylated and Mono-pegylated High Affinity (Super Active) Rat Leptin Antagonist (SRLA and PEG-SRLA)

The template used for super mutant construction was RLA mutant (L39A/D40A/F41A) [20] in the prokaryotic expression vector pMon [21]. The expression vector was modified with the Stratagene QuikChange mutagenesis kit according to the manufacturer’s instructions using 2 complementary primers: the sense primer 5′-CAATTGTCACCAGGAT**T**AAT **CTG**ATTTCACACACGCAG-3′ (the mutated bases are in bold and VspI restriction site is underlined), and the antisense primer 5′-CTGCGTGTGTGAAATCAGATTAATCCTGGTGACAATTG-3′. The procedure was identical to that described recently [22]. Two colonies were sequenced and confirmed to contain the mutation without any undesired misincorporation of nucleotides. Mon105 competent cells were then transformed with the mutated plasmid and used for expression. The mutated protein (D23L/L39A/D40A/F41A) with an extra Met-Ala (Met is cleaved by the bacteria) at its N terminus was expressed in 2.5 liters of culture, upon induction with nalidixic acid [21] and grown for an additional 4 h. Inclusion bodies (IBs) were then prepared as described previously [21] and frozen. Subsequently, IBs obtained from 5 liters of bacterial culture were solubilized in 600 ml of 4.5 M urea and 40 mM Tris base containing 0.1 mM cysteine and adjusted to pH 11.3 with NaOH. After 2 h of stirring at 4°C, three volumes of 0.67 M arginine were added to a final concentration of 0.5 M, and stirring was continued for an additional 1.5 h. Then the solution was dialyzed against 10 liters of 10 mM Tris-HCl, pH 10, for 60 h, with six external solution exchanges and applied at maximal flow rate (400–500 ml/h) onto DEAE-cellulose column (DE-52, Whatman Co.) of 30-ml bead volume, pre-equilibrated with 10 mM Tris base adjusted to pH 10. The absorbed protein was eluted in a stepwise manner using increased concentration of NaCl in the same buffer. The monomeric fraction was eluted with 50 mM yielding 350–400 mg of pure monomeric SRLA. The pooled fraction was dialyzed against NaHCO_3_ to ensure a 4∶1 protein-to-salt ratio, and lyophilized. The pegylation was carried out as described before for superactive ovine leptin antagonist [22] but the mono-pegylated SRLA (PEG-SRLA) was eluted from the SP-Sepharose column with 75 mM NaCl, dialyzed against NaHCO_3_ to ensure a 3∶1 protein-to-salt ratio, and lyophilized. The overall yield from 350 mg of PEG-SRLA was 100 to 120 mg.

Purity check by SDS-PAGE and analytical gel filtration along with testing the binding capacity and inhibitory activity of leptin-induced proliferation in Baf/3 cells were carried out exactly as described before [22]. BAF/3 cells stably transfected with chimeric receptor composed of an extracellular and transmembrane domain of the murine leptin receptor fused to the intracellular domain of the human βc receptor, a common receptor subunit present in several cytokine receptors.

### Animals and Experimental Groups

The study was performed in adult male Wistar rats weighing 213±4 g before the experiment. After acclimation, the animals were divided into the following groups (n = 6 each, Table 1): (1) control, fed standard rat chow (68% calories from carbohydrates, 20% protein and 12% fat) *ad libitum* for 4 weeks, (2) leptin-treated group fed standard chow for 4 weeks in which hyperleptinemia was induced by administration of exogenous leptin for the last 7 days, (3) group receiving high-calorie palatable diet for 4 weeks (obese group), (4) group fed highly palatable diet for 4 weeks and receiving PEG-SRLA during the last week, (5) group fed standard chow for 4 weeks and receiving PEG-SRLA during the last week. High-calorie diet consisted of standard chow combined 1∶1 (wt/wt) with a liquid diet containing equal amounts of sucrose, glucose, whole milk powder and soybean powder suspended in tap water [23]. The composition of this diet was similar to standard chow (66% calories from carbohydrates, 20% from protein, and 14% from fat). Animals in all groups were at the same age at the end of experiment. The study protocol was reviewed and approved by the Bioethical Committee of the Lublin Medical University.

**Table 1:** Diets and treatments applied in different experimental groups.

Experimental group	Diet (weeks 1–4)	Treatment during the 4^th^ week
Control	Standard	–
Leptin-treated	Standard	Leptin (0.25 mg/kg twice daily)
Obese	High-calorie	–
Obese+PEG-SRLA	High-calorie	PEG-SRLA (7 mg/kg every other day)
PEG-SRLA	Standard	PEG-SRLA (7 mg/kg every other day)

Hyperleptinemia was induced by administration of recombinant rat leptin. Leptin was injected at a dose of 0.25 mg/kg twice daily between 7 and 8 AM and between 7 and 8 PM into the subcutaneous adipose tissue in the interscapular region. PEG-SRLA was administered for the last week of the 4-week experiment in rats fed regular or high-calorie diet. PEG-SRLA was injected every other day at 7 mg/kg ip. between 7.00 and 8.00 AM. Animals were anesthetized to perform vascular reactivity experiments 24 hours after the last PEG-SRLA dose or 6 hours after the last leptin injection. All together four doses of PEG-SRLA were administered to each rat. Food was withdrawn from the cages 6 hours before sacrifice to induce the fasting state.

### Measurement of Vascular Tone

Animals were anesthetized with thiopental (50 mg/kg ip.). Abdominal cavity was opened and blood was collected from the abdominal aorta for the measurement of leptin, insulin, glucose and lipid profile. First and second-order mesenteric artery branches (internal diameter 250–300 µm) were gently dissected, placed in HEPES- buffered saline solution containing 142 mM NaCl, 4.7 mM KCl, 1.2 mM MgSO_4_, 2.5 mM CaCl_2_, 1.2 mM KH_2_PO_4_, 5.5 mM glucose and 10 mM HEPES (pH 7.4) saturated with 95%O_2_/5% CO_2_ gas mixture, cleaned of adherent connective and adipose tissue and cut into 2 mm segments. In some experiments endothelium was removed from the vessel by repeatedly passing stainless steel cannula of appropriate size through the vessel lumen. Integrity of the vessel after denudation was verified by measuring contractility induced by 60 mM KCl; only vessels with contractility comparable to those of endothelium-intact rings were used. Destruction of the endothelium was confirmed by loss of the relaxation response to acetylcholine (1 µM). Arterial segments were mounted into the multiwire myograph system DMT 610 (Danish Myo Technology, Aarhus, Denmark) and kept in HEPES-buffered saline solution bubbled with 95%O_2_/5%CO_2_ (pH 7.4) at 37°C throughout the experiment. Vessels were then stretched to 90% of diameter that would be obtained if the artery was subjected to 100 mmHg internal pressure, which produced maximal tension responses. Vessels were initially exposed to 60 mM KCl to measure their maximal contractility. After the 30-min washout period, concentration–response curve to the α-adrenergic agonist phenylephrine was constructed by its cumulative addition (1 nM–10 µM). Tension was expressed as the percentage of tension obtained with 60 mM KCl. After washout, segments were contracted to 75% of maximal tension with appropriate concentration of phenylephrine and then leptin was added at increasing concentrations (0.01–500 ng/ml). Relaxation in response to leptin was expressed as a percentage of sustained phenylephrine-induced contraction. If the effect of leptin was examined in the presence of inhibitors of specific pathways, respective inhibitors were administered 15 min before the first dose of leptin. The effective concentrations causing 50% of maximal response (EC_50_) and the maximal relaxations (R_max_) were calculated for each ring by nonlinear regression, and mean ± SD values calculated for respective experimental groups are presented in tables. Data presented on figures are relaxations induced by individual leptin concentrations (mean ± SD).

### Cystathionine γ-lyase (CSE) Activity in Mesenteric Arteries

CSE activity was measured by two methods: (i) using L-cystathionine as the substrate, i.e. decomposition of L-cystathionine to L-cysteine in the transsulfuration pathway of homocysteine metabolism, (ii) using L-cysteine as the substrate, that is desulfhydration of L-cysteine to H_2_S. CSE activity toward cystathionine was measured by a method of Stipanuk et al. [24] as recently described [25]. Vascular segments were homogenized in 50 mM ice-cold potassium phosphate buffer (pH 7.4) and centrifuged at 10 000 g for 20 min. Supernatant (50 µl) was added to 1 ml reaction medium containing 100 mM potassium phosphate buffer (pH 7.4), 4 mM l-cystathionine, 0.125 mM pyridoxal 5′-phosphate, 0.32 mM NADH and 1.5 U/ml lactate dehydrogenase. Decrease in absorbance at 340 nm, reflecting consumption of NADH during lactate dehydrogenase-catalyzed reduction of 2-oxobutyrate (produced from cystathionine by CSE) to 2-hydroxybutyrate, was recorded. Blank samples without cystathionine were subtracted and CSE activity was calculated from the linear portion of the graph and is expressed in nmol/min per mg protein.

In addition, CSE activity toward L-cysteine (desulfhydration reaction) was measured as the H_2_S formation [25]. Supernatant (0.25 ml) was incubated for 90 min at 37°C in sealed tubes in the presence of 2 mM l-cysteine and 2 mM pyridoxal 5′-phosphate. After the incubation, 0.125 ml of 20% trichloroacetic acid was injected to the tubes to stop the reaction, followed by 0.125 ml of 15 mM zinc acetate and 0.5 ml of borate buffer (pH 10.0). The tubes were incubated at 37°C for additional 60 min. Subsequently, the reaction solution was mixed with 0.5 ml of 20 mM N,N-dimethyl-p-phenylenediamine sulfate in sulfuric acid and 20 µl of 30 mM FeCl_3_. After 30 min, the sample was centrifuged at 5000 × *g* for 3 min and the absorbance at 670 nm was measured. The absorbance of blank sample, to which trichloroacetic acid was added before incubation, was subtracted from the absorbance of a test sample, and hydrogen sulfide concentration was calculated against a calibration curve based on different concentrations (3.12–250 µM) of NaHS. Results were expressed as pmol H_2_S generated during 1 min per mg of protein (pmol/min/mg).

### Other Assays

Plasma insulin and leptin concentrations were assayed by EIA method using Rat Insulin EIA Kit (SPIbio, Massy, France) and Leptin Enzyme Immunoassay Kit (Cayman Chemical), respectively. Plasma triglycerides, total cholesterol, HDL-cholesterol and glucose were measured by commercially available kits (Alpha Diagnostics, Warsaw, Poland).

### Reagents

Recombinant rat leptin was obtained from R&D Systems. Chromium(III) mesoporphyrin IX was purchased from Frontier Scientific (Logan, Utah, USA). GYY4137 was synthesised and chemically characterized in house [26], [27]. Other reagents were obtained from Sigma-Aldrich.

### Statistical Analysis

Data are presented as mean ± SD from 6 rats/group. Between-group comparisons were performed by two-tailed Student t-test or ANOVA followed by Tukey post-hoc test for 2 and >2 groups, respectively. When the same vascular preparation was examined under two different conditions (for example with and without the inhibitor), t-test for related variables was used. P<0.05 was considered significant.

## Results

### Preparation and Characterization of SRLA and PEG-SRLA

**Figure 1:**
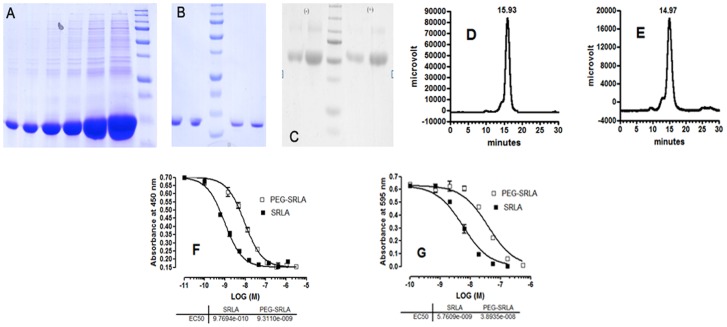
Preparation and characteristics of superactive rat leptin antagonist. (**A**) Inclusion bodies from 2.5 L fermentation culture were prepared and resuspended in 100 ml of DDW. Aliquots (corresponding to 0.8, 1.6, 3.2, 4.0, 8.0 and 12.0 µl per lane, from left to right) were separated by 15% SDS-PAGE in presence of β-mercaptoethanol. The molecular mass markers from the bottom up (last lane on the right) are (in kDa): 10, 15, 20, 25, 37, 50, 75, 100, 150 and 250. (**B**) SDS-PAGE (15%) of purified SRLA run after lyophilization in the absence (lanes 1–2 from the left) or presence (lanes 4–5 from the left) of β-mercaptoethanol (ME) at 2 concentrations: lanes 1 and 4–5 µg, lanes 2 and 5–10 µg. Lane 3– molecular weight markers (see above). (**C**) SDS-PAGE (10%) of purified PEG-SRLA run after lyophilization in the absence (lanes 1–2 from the left) or presence (lanes 4–5 from the left) of β-mercaptoethanol (ME) at 2 concentrations: lanes 1 and 4–5 µg, lanes 2 and 5–10 µg. Lane 3– molecular weight markers (see above); (**D**) Gel-filtration analysis of the purified SRLA on analytical Superdex 75 column pre-equilibrated with TN buffer, pH 8. The main peak with retention time of 15.93 min corresponds to monomer and the preceding small shoulder to dimer. (**E**) Gel-filtration analysis of the purified PEG-SRLA on analytical Superdex 200 column pre-equilibrated with TN buffer, pH 8. The main peak with retention time of 14.93 min corresponds to mono-pegylated PEG-SRLA and the preceding small shoulder to double-pegylated PEG-SRLA. To estimate the molecular mass shown in (D) and (E) the columns were calibrated with BSA (66 kDa), rat CNTF (22 kDa) and human leptin (16 kD); (**F**) Competitive non-radioactive receptor-binding assay of SRLA and PEG-SRLA. Binding of biotinylated human leptin to immobilized human leptin binding domain (hLBD) consisting of the amino acids 428–635 of human leptin receptor [51] was performed in the presence of the indicated protein SRLA or PEG-SRLA concentrations. The experiment was carried out in triplicates and the results are presented as mean ± SEM. As the variations in this assay was very small the error bars are not seen; (**G**) Biological activity of SRLA and PEG-SRLA. The experiment was performed in BAF/3 cells stably transfected with the chimeric leptin receptor construct consisting of the extra-cellular and transmembrane domain of the murine leptin receptor with the intracellular domain of the human βc receptor. Synchronized cells were grown for 48 h in the presence of rat leptin (50 pg/well) and various concentrations of SRLA or PEG-SRLA. The number of cells was then determined by the MTT method. In both bioassays the experiment was carried out in triplicates and the results are presented as mean ± SEM. Detailed description of the binding experiments and the bioassay is provided in our former paper [28].

Preliminary experiments aiming at expression of SRLA conducted in four *E. coli* clones indicated strong expression in most of them (not shown). The best expressing clone was picked for large scale expression. The inclusion bodies prepared as described before [28] contained highly purified unfolded SRLA (Fig. 1A). After refolding and dialysis SRLA was purified by single-step anion-exchange chromatography on DEAE column as described in Methods. The fractions containing pure monomer eluted with 50 mM NaCl from the DEAE –cellulose column were pooled, dialyzed against NaHCO_3,_ pH 8, at a 4∶1 (w/w) protein:salt ratio and lyophilized or filter-sterilized and stored at 4°C till pegylation. The purity and homogeneity of the purified leptin antagonist were documented by two independent methods. SDS-PAGE under reducing conditions yielded only one band of ∼ 16 kDa, under both reducing and nonreducing conditions (Fig. 1B). Gel filtration at pH 8 under native conditions yielded a single monomeric peak consisting of over 95% monomer, corresponding to a molecular mass of ∼ 15 to 16 kDa (Fig. 1D). Specific extinction coefficients at 280 nm for a 0.1% solution, assuming an extra Ala at the N-terminus, were calculated according to Pace et al. [29] yielding the value of 0.200. Pegylation of the monomeric SRLA obtained from 5 l of fermentation culture yielded mono-pegylated (90%) with small (10%) double pegylated product. Its purity is shown in Figs. 1C and 1E. The binding properties and the biological activity of the purified SRLA and PEG-SRLA was tested by binding to human leptin receptor binding domain and in Baf/3 bioassay (Figs. 1F and 1G), showing high similarity to the activities showed previously for similar mouse and ovine superactive leptin antagonists [22], [28]. The stability of SRLA and PEG-SRLA in solution was tested at 4°C and 37°C, stored at both temperatures as sterile 2 mg/ml solutions for at least 14 days at pH 8 without undergoing any changes in their monomeric content and retaining their activity in the Baf/3 bioassay.

### Characteristics of Experimental Groups

**Figure 2:**
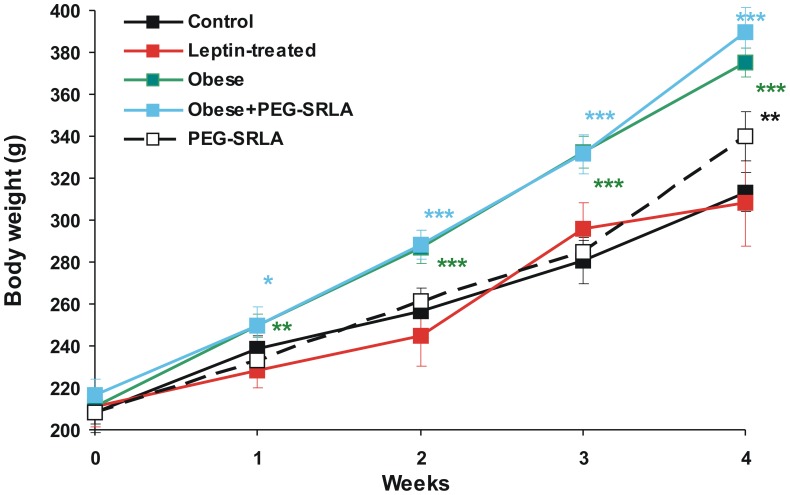
Body weight of rats in different experimental groups. *p<0.05, **p<0.01, ***p<0.001 vs. control group at the respective time point (ANOVA and Tukey test).

Body weight (monitored weekly) increased progressively in all groups of rats throughout the duration of the study. Since the end of the first week, body weight of both groups fed the highly palatable diet was significantly higher than of animals fed regular diet (Fig. 2). Terminal body weight after 4 weeks of feeding was higher in obese, obese+PEG-SRLA and PEG-SLRA-treated groups than of control group by 19.8%, 24.4%,and 8.4% respectively. Leptin treatment had no effect on terminal body weight (Fig. 2).

The weight gain in the last week of the experiment was higher in PEG-SRLA-treated than in control group (54.7±4.5 g vs. 32.5±3.4 g, p<0.001). The mean weight gain in PEG-SRLA-treated obese rats was also higher than in vehicle-treated obese rats (58.2±7.3 g vs. 43.0±3.9 g) but this difference was not statistically significant due to the large variability in the PEG-SRLA treated obese group. Weight gain of leptin-treated rats (12.0±7.0 g) was significantly lower (p<0.001) than in the control group.

Plasma leptin concentration was significantly higher in leptin-treated and obese rats than in control group (Table 2). Because anti-leptin antibodies used in the ELISA assay cross-react with leptin antagonist, leptin levels were not measured in PEG-SRLA-treated groups. Plasma insulin, glucose and lipids did not differ between the groups (Table 2).

**Table 2:** Metabolic characteristics of animals in different experimental groups.

Group	Leptin (ng/ml)	Insulin (ng/ml)	Glucose (mM)	Triglycerides (mM)	Total cholesterol (mM)	HDL-cholesterol (mM)
Control	3.62±0.23	2.38±0.14	5.75±0.50	0.90±0.10	2.06±0.14	1.46±0.10
Leptin-treated	13.60±0.89***	2.22±0.11	5.84±0.53	0.86±0.07	2.00±0.09	1.43±010
Obese	11.77±0.64***	2.51±0.18	5.70±0.55	0.99±0.15	2.02±0.11	1.47±0.12
Obese PEG-SRLA-treated	–	2.51±0.32	6.20±0.31	1.04±0.17	2.01±0.12	1.45±0.09
PEG-SRLA-treated	–	2.55±0.14	5.58±0.41	1.00±0.18	2.01±0.14	1.55±0.13

*** p<0.001 vs. control group (ANOVA and Tukey test).

### Vascular Effect of Leptin and its Mechanism

**Figure 3:**
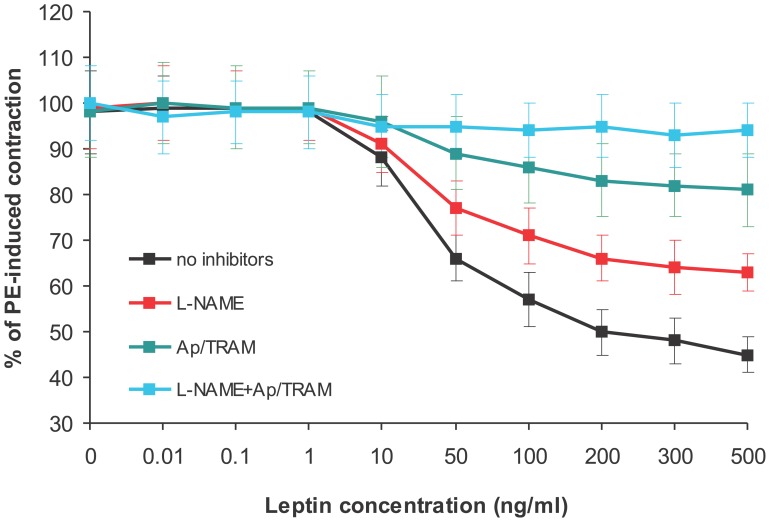
Leptin-induced relaxation of phenylephrine (PE)-preconstricted mesenteric artery rings of control rats with intact endothelium in the absence of inhibitors and in the presence of NO synthase inhibitor, L-NAME, or inhibitors of small and intermediate-conductance Ca^2+^-activated K^+^ channels, apamin (Ap) and TRAM-34 (TRAM), respectively, which together block EDHF-dependent response.

No statistically significant difference in maximal contraction induced by KCl was observed between groups. Phenylephrine (PE) consistently constricted mesenteric artery rings to the similar extent in all experimental groups (not shown). In the control group, leptin induced concentration-dependent relaxation of PE-preconstricted mesenteric artery rings with intact endothelium (Fig. 3). The effect of leptin was attenuated by the NO synthase inhibitor, L-NAME (100 µM), as well as by the inhibitors of small- and intermediate-conductance calcium-activated potassium channels, apamin (5 µM) and TRAM-34 (1 µM), compounds well-known to inhibit EDHF-dependent vasorelaxation [30]. The combination of L-NAME and apamin/TRAM-34 almost completely abolished leptin-induced vasorelaxation (Fig. 3). In contrast, the cyclooxygenase (COX) inhibitor, indomethacin (10 µM) did not attenuate the effect of leptin (not shown). These results suggest that leptin induced vasorelaxation in NO- and EDHF-dependent but COX-independent manner. Leptin also relaxed mesenteric artery rings with denuded endothelium but this effect was much less potent (R_max_ = 11±3%, EC_5O_ = 44±6 ng/ml). The endothelium-independent component was not further examined in this study.

### Vascular Effect of Leptin in Different Experimental Groups

**Figure 4:**
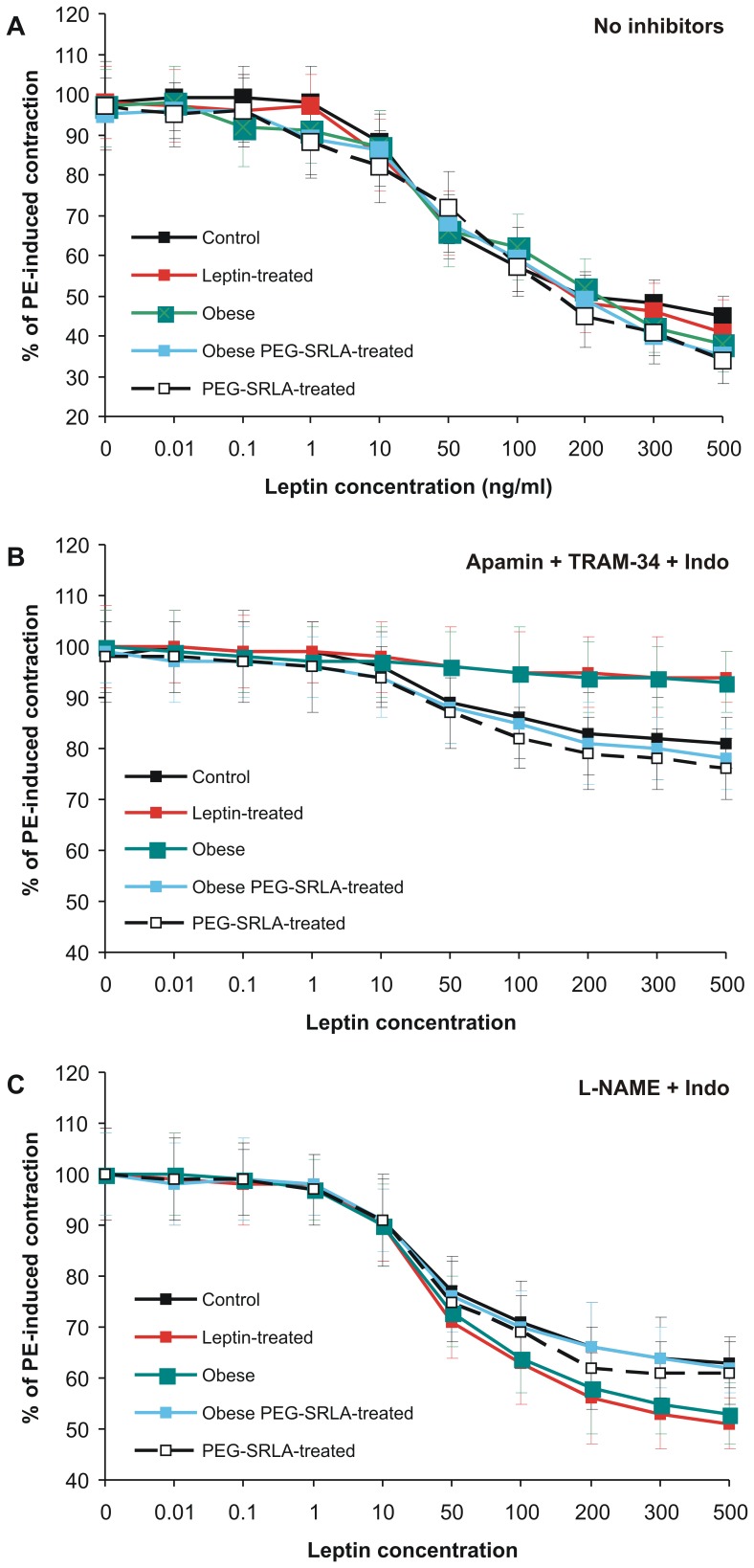
Leptin-induced relaxation of PE-preconstricted mesenteric artery rings in different experimental groups. Effect of leptin was examined without inhibitors (A), in the presence of apamin, TRAM-34 and indomethacin (Indo; NO-dependent vasorelaxation, B) or in the presence of NO synthase inhibitor, L-NAME, and indomethacin (EDHF-dependent vasorelaxation, C).

In the subsequent studies, we examined NO- and EDHF-mediated components of leptin-induced endothelium-dependent vasorelaxation as the effects persisting in the presence of apamin/TRAM-34 plus indomethacin and L-NAME plus indomethacin, respectively. In the absence of any inhibitors, leptin relaxed PE-preconstricted mesenteric artery rings in all groups of rats to the similar extent (Fig. 4A). NO-dependent vasodilatory effect of leptin was significantly impaired in the leptin-treated and obese groups but in the latter group was restored by PEG-SRLA treatment (Fig. 4B). PEG-SRLA alone administered to animals fed the regular chow had no effect on the NO-dependent vasodilatory effect of leptin (Fig. 4B). Mathematical analysis revealed that maximal NO-dependent dilatory effect of leptin was impaired in the leptin-treated and obese groups whereas EC_50_ did not change (Table 3). In contrast, the EDHF-dependent component of the vasodilatory effect of leptin was enhanced in the leptin-treated and obese groups and, again, this was associated with the increase in R_max_ but not with any changes of EC_50_ (Fig. 4C, Table 3). PEG-SRLA treatment decreased EDHF-mediated vasodilatory effect of leptin in the obese group to the level observed in the control group. However, PEG-SRLA alone administered to lean animals had no effect. These results suggest that NO-dependent and EDHF-dependent portions of leptin-induced vasorelaxation are impaired and enhanced, respectively, by either chronic leptin treatment or obesity, and in obese animals both these changes are abolished by the leptin antagonist.

**Table 3:** Leptin-induced relaxation of phenylephrine-preconstricted mesenteric artery rings in different experimental groups.

	Total		NO-dependent		EDHF-dependent	
	R_max_	EC_50_	R_max_	EC_50_	R_max_	EC_50_
Control	59.0±4.9	38.3±7.8	21.0±4.5	48.5±11.9.	40.1±2.5	36.1±7.0
Leptin-treated	60.4±8.4	46.8±5.4	6.4±0,8***	36.4±11.2	53.4±5.0**	43.5±11.5
Obese	59.4±9.3	42.8±9.0	6.7±1.6***	38.8±13.7	51.2±8.5*	43.6±11.6
Obese +PEG-SRLA	59.7±8.6	44.9±13.0	20.9±3.6	67.2±13.9	40.7±3.0	36.3±6.5
PEG-SRLRA	60.2±8.4	43.5±13.7	20.9±4.4	59.3±12.3	39.9±5.8	36.1±6.9

NO-dependent vasorelaxation was measured in the presence of apamin (5 µM), TRAM-34 (1 µM) and indomethacin (10 µM), and EDHF-dependent vasorelaxation was examined in the presence of L-NAME (100 µM) plus indomethacin (10 µM). Maximal relaxation (R_max_) was calculated as the leptin-induced percent decrease in tension developed in response to phenylephrine. EC_50_– leptin concentration (ng/ml) which induced a half-maximal relaxation of PE-preconstricted segments. R_max_ and EC_50_ values were calculated for each individual vascular preparation and data presented in the table are mean ± SD from 6 animals per group.

* p<0.05,

** p<0.01,

*** p<0.001 vs. control group (ANOVA and Tukey test).

### Mechanism of EDHF-mediated Vascular Effect of Leptin in Rat Mesenteric Artery

**Figure 5:**
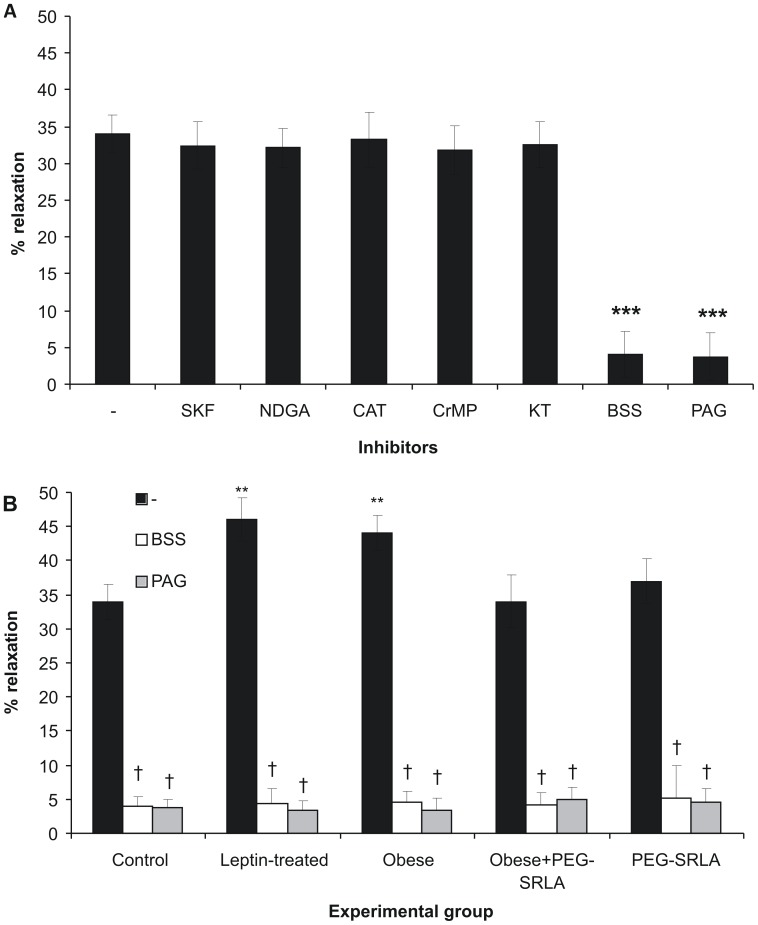
Effect of inhibitors of different EDHF mechanisms on leptin-induced vasorelaxation. **A:** Mesenteric artery segments of control rats were preconstricted with phenylephrine, and the vasodilating effect of leptin (200 ng/ml) was examined in the presence of L-NAME and indomethacin without other inhibitors or after addition of cytochrome P450 inhibitor SKF 525A (SKF, 10 µM), lipoxygenase inhibitor nondihydroguaiateric acid (NDGA, 20 µM), H_2_O_2_ scavenger PEG-catalase (CAT, 250 U/ml), heme oxygenase inhibitor Cr(III) mesoporphyrin IX (CrMP, 10 µM), protein kinase G inhibitor KT5823 (KT, 1 µM), H_2_S scavenger bismuth (III) subsalicylate (BSS, 10 µM) or cystathionine γ-lyase inhibitor D,L-propargylglycine (PAG, 1 mM). ***p<0.001 vs. preparation treated only with L-NAME and indomethacin (Student t-test for related variables) **B:** Effect of leptin (200 ng/ml) on PE-preconstricted mesenteric artery segments was examined in different experimental groups in the presence of L-NAME and indomethacin (black bars), L-NAME, indomethacin and BSS (white bars) or L-NAME, indomethacin and PAG (grey bars). **p<0.01 vs. control group, †p<0.001 vs. respective group not treated with either PAG or BSS.

EDHF has been demonstrated to be accounted for by various mediators such as cytochrome P450-dependent (epoxyeicosatrienoic acids, EETs) or 15-lipoxygenase-dependent (11,12,15-trihydroxyeicosatrienoic acids – THETAs and 15-hydroxy-11,12-epoxyeicosatrienoic acids – HEETAs) arachidonate derivatives, H_2_O_2_, heme oxygenase-derived carbon monoxide (CO), or C-type natriuretic peptide (CNP) [31]. To address the question if any of these factors were involved in leptin-induced vasorelaxation, we examined the EDHF-dependent effect of leptin in the presence of respective inhibitors. However, cytochrome P450 inhibitor (SKF 525A, 10 µM), lipoxygenase inhibitor, nondihydroguaiateric acid (NDGA, 20 µM), H_2_O_2_ scavenger (PEG-catalase, 250 U/ml), heme oxygenase inhibitor (Cr(III) mesoporphyrin IX, 10 µM) and protein kinase G inhibitor which blocks natriuretic peptide signaling (KT5823, 1 µM) had no effect on leptin-induced relaxation of PE-preconstricted mesenteric artery. In contrast, the H_2_S scavenger, bismuth (III) subsalicylate (BSS, 10 µM) [32] or CSE inhibitor, D,L-propargylglycine (PAG, 1 mM), almost completely abolished the EDHF-dependent portion of leptin-induced vasorelaxation (Fig. 5A). In contrast, neither BSS nor PAG had any effect on NO-dependent portion of leptin-induced vasorelaxation (not shown). Similarly, BSS or PAG abolished EDHF-mediated portion of leptin-induced vasorelaxation in leptin-treated, obese, obese PEG-SRLA-treated and lean PEG-SRLA-treated rats, suggesting that the mechanism of leptin-induced EDHF remains H_2_S-dependent in all groups (Fig. 5B).

### Effect of H_2_S Donor, GYY4137, on Phenylephrine-preconstricted Mesenteric Artery Rings

**Figure 6:**
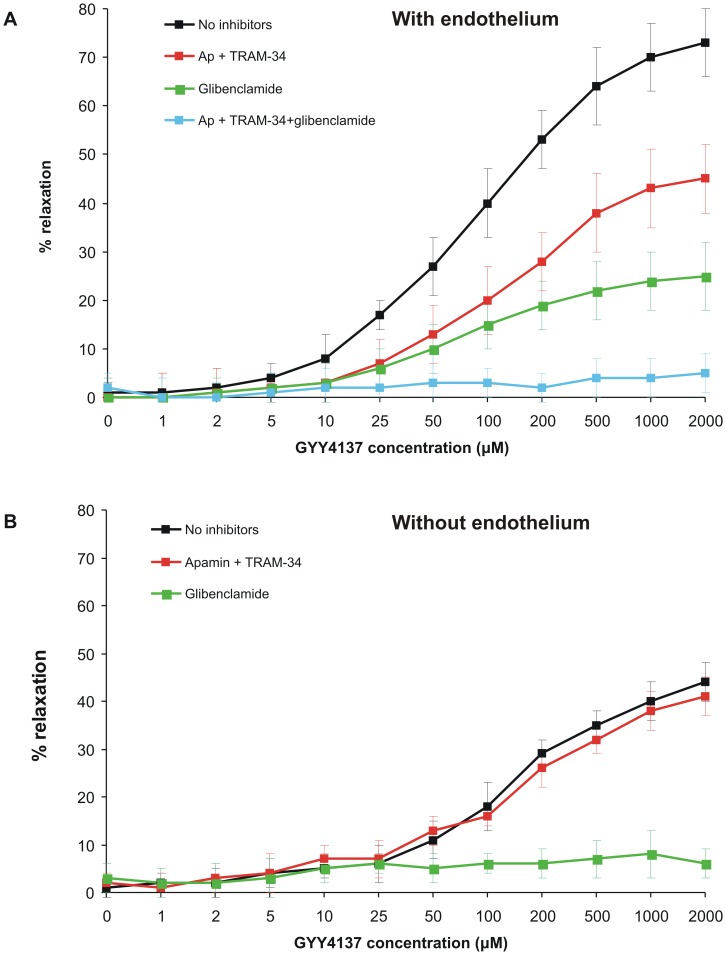
Relaxing effect of H_2_S donor, GYY4137, on phenylephrine-preconstricted mesenteric artery segments of control rats with intact (A) or removed (B) endothelium examined in the absence of K^+^ channel inhibitors (black), in the presence of SK_Ca_ and IK_Ca_ inhibitors (apamin and TRAM-34, respectively, red), in the presence of K_ATP_ channel inhibitor, glibenclamide (green) or in the presence of all three inhibitors (blue).

The synthetic H_2_S donor, GYY4137 [26], [27], relaxed PE-preconstricted mesenteric artery segments with intact endothelium in a concentration-dependent manner (Fig. 6A). The effect of GYY4137 was partially attenuated by apamin and TRAM-34 or by the K_ATP_ channel antagonist, glibenclamide (10 µM), and completely abolished by the mixture of these three K^+^ channel inhibitors (Fig. 6A). Apamin and TRAM-34 markedly reduced maximal relaxation and significantly increased EC_50_ (i.e. reduced the sensitivity to vasodilating effect of GYY4137). Glibenclamide decreased R_max_ value and the mean EC_50_ but the latter effect was not significant (Table 4). In sharp contrast, decomposed GYY4137, dissolved 12 hours prior to use to exhaust H_2_S [27], had no effect on mesenteric arteries (not shown).

**Table 4:** GYY4137-induced relaxation of phenylephrine-preconstricted mesenteric artery rings.

	With endothelium		Without endothelium	
	R_max_ (%)	EC_50_ (µM)	R_max_ (%)	EC_50_ (µM)
No inhibitors	77.3±5.6	96.6±17.6	47.2±4.4†††	135.1±7.0†††
Apamin+TRAM-34	47.7±7.4***	140.1±12.6***	45.4±5.0	146.1±16.6
Glibenclamide	29.4±3.8***	79.9±17.4	-	-

Effect of increasing concentrations of GYY4137 on mesenteric artery segments with intact or removed endothelium was examined in the absence or in the presence of K^+^ channel inhibitors and R_max_ and EC_50_ values were calculated for each preparation (n = 6/group).

*** p<0.001 vs. preparation without K^+^ channel inhibitors (Student t-test for related variables),

††† p<0.001 vs. segments with intact endothelium (Student t-test for unrelated variables). Because glibenclamide almost completely abolished the effect of GYY4137 in endothelium-denuded segments, R_max_ and EC_50_ could not be calculated.

GYY4137 also relaxed endothelium-denuded mesenteric artery rings preconstricted with phenylephrine but to a lesser extent than endothelium-intact segments (Fig. 6B, Table 4). The effect of GYY4137 on endothelium-denuded arteries was not altered by apamin and TRAM-34 but was completely abolished by glibenclamide. GYY4137-induced relaxation measured in the presence of apamin+TRAM-34 did not differ between segments with intact and with removed endothelium. These results suggest that when H_2_S is released slowly (e.g. from GYY4137), it relaxes phenylephrine-preconstricted mesenteric arteries through the mechanisms involving endothelial SK_Ca_ and IK_Ca_ channels and smooth muscle cell K_ATP_ channels.

### CSE Activity

CSE activity toward L-cystathionine was similar in all groups of animals (control: 2.97±0.36 nmol/mg/min, leptin-treated: 2.62±0.39 nmol/mg/min, obese: 2.51±0.29 nmol/mg/min, obese PEG-SRLA-treated: 3.11±0.41 nmol/mg/min, lean PEG-SRLA-treated: 3.25±0.44 nmol/mg/min). In addition, CSE activity toward L-cysteine (H_2_S formation from L-cysteine by tissue homogenates) did not differ between groups (control: 89.2±9.7 pmol/mg/min, leptin-treated: 82.1±8.4 pmol/mg/min, obese: 80.3±9.2 pmol/mg/min, obese SLRA-treated: 86.0±8.4 pmol/mg/min, lean SLRA-treated: 90.4±7.9 pmol/mg/min). These results suggest that the ability of mesenteric vessels to synthesize H_2_S was similar in all experimental groups.

### Leptin-induced Vasorelaxation in the Presence of SQR Inhibitor

**Figure 7:**
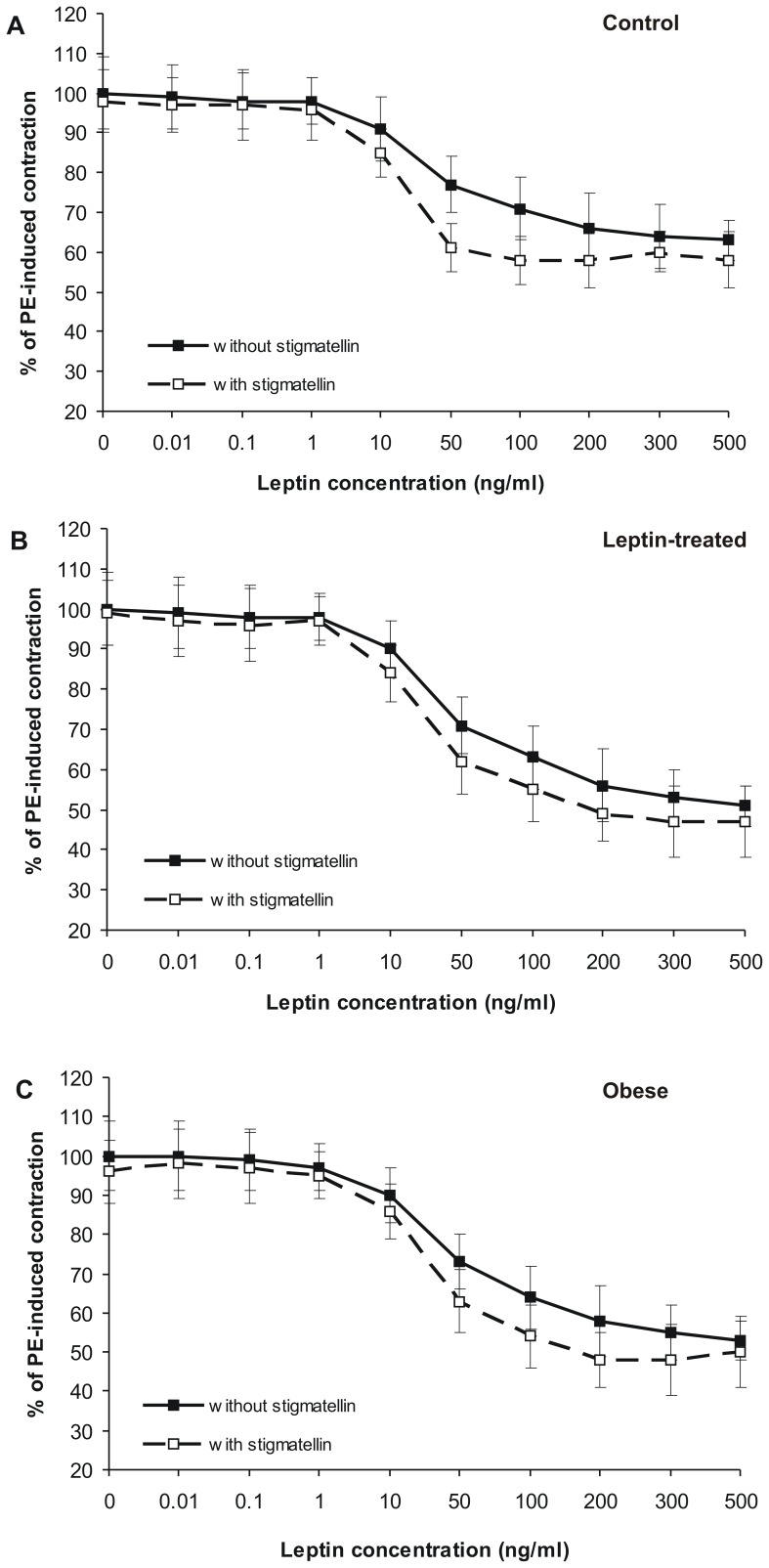
Effect of SQR inhibitor, stigmatellin, on EDHF-mediated leptin-induced relaxation of mesenteric artery segments. Concentration-dependent effect of leptin on PE-preconstricted rings from control (A), leptin-treated (B) and obese (C) rats was examined in the presence of L-NAME+indomethacin. Segments were examined in the absence (black squares, continuous line) or in the presence of 3 µM stigmatellin (white squares, broken line).

H_2_S signaling in tissues is regulated not only by its synthesis but also by mitochondrial oxidation. We hypothesized that enhanced leptin induced EDHF/H_2_S-mediated vasorelaxation in leptin-treated and obese groups could be accounted for by reduced H_2_S oxidation. To address this issue, we examined leptin-induced vasorelaxation in the presence of stigmatellin which inhibits sulfide:quinone oxidoreductase (SQR) – the first enzyme of mitochondrial H_2_S oxidation [33]. Stigmatellin alone (0.1–10 µM) had no effect on PE-preconstricted mesenteric artery segments (not shown). However, stigmatellin (3 µM) augmented EDHF-dependent portion of leptin-induced vasorelaxation in control, leptin-treated and obese rats (Fig. 7). Due to insufficient amount of material from PEG-SRLA-treated rats, these experiments were not performed in these groups Although the difference was not large, leptin at all concentrations induced more marked relaxation of mesenteric artery segments in the presence than in the absence of stigmatellin. In addition, mathematical analysis revealed that stigmatellin reduced EC_50_ in all groups of rats (Table 5). Although stigmatellin also tended to increase maximal relaxation induced by leptin, this effect was small and significant only in vessels from leptin-treated animals. However, stigmatellin did not eliminate the differences in leptin-induced relaxation between groups. Maximal EDHF-dependent relaxing effect of leptin remained higher in leptin-treated and obese compared to the control group (Table 5). In contrast, stigmatellin had no effect on NO-dependent portion of leptin-induced vasorelaxation (not shown). These data suggest that although mitochondrial H_2_S oxidation affects leptin-induced H_2_S signaling in the vascular wall, it is not responsible for enhanced effect of leptin in leptin-treated or obese rats.

**Table 5:** EDHF-mediated leptin-induced relaxation of phenylephrine-preconstricted mesenteric artery rings in the absence and in the presence of stigmatellin.

	Without stigmatellin		With stigmatellin	
Group	R_max_ (%)	EC_50_ (ng/ml)	R_max_ (%)	EC_50_ (ng/ml)
Control	40.1±2.5	36.1±6.9	42.3±3.0	24.9±3.0**
Leptin-treated	53.4±5.0††	43.5±11.5	58.1±3.6*** †††	34.9±9.1*
Obese	51.2±8.5†	43.6±11.6	54.5±7.0††	33.8±8.6**

* p<0.05,

** p<0.01,

*** p<0.001 vs. respective segment not treated with stigmatellin (Student t-test for related variables),

† p<0.05,

†† p<0.001,

††† p<0.001 vs. control group (ANOVA and Tukey post-hoc test).

### GYY4137-induced Vasorelaxation in Leptin-treated and Obese Rats

**Figure 8:**
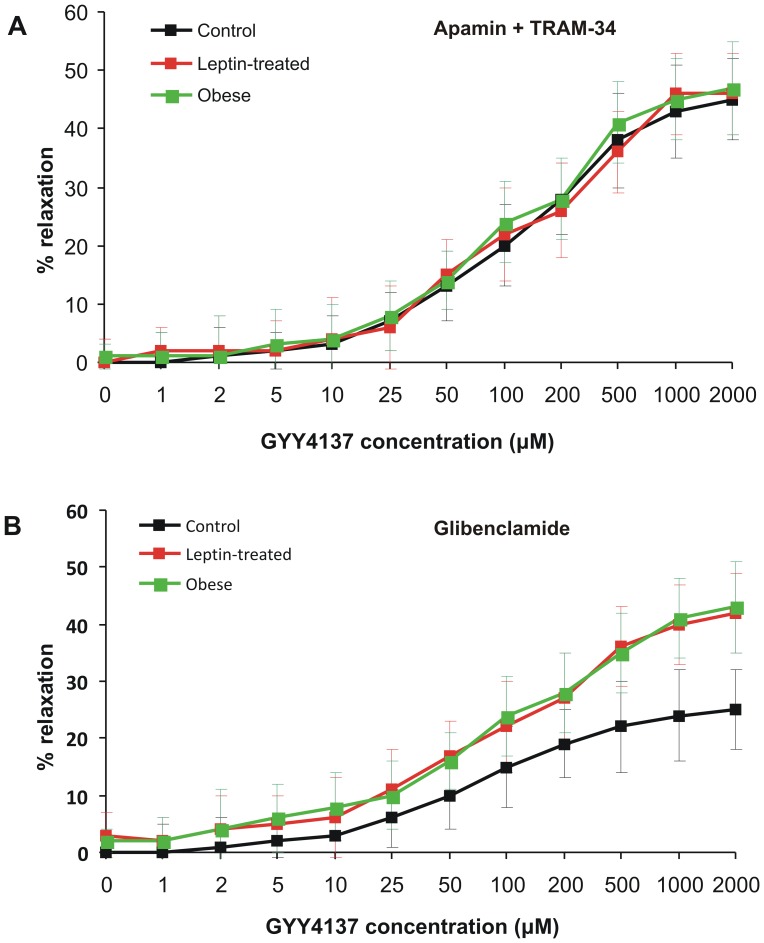
Effect of GYY4137 on PE-preconstricted mesenteric artery rings from control (black), leptin-treated (red) and obese (green) rats in the presence of apamin and TRAM-34 (A) or glibenclamide (B).

Finally, we examined if the sensitivity of blood vessels to H_2_S is altered in leptin-treated and obese rats. We examined GYY4137-induced relaxation of PE-preconstricted mesenteric artery rings with intact endothelium in the presence of apamin and TRAM-34 or glibenclamide, which reflect K_ATP_ channel-dependent and SK_Ca_/IK_Ca_ channel-dependent mechanisms, respectively. The effect of GYY4137 on apamin+TRAM-34 treated segments was similar in all groups (Fig. 8A). In contrast, the effect of H_2_S donor on glibenclamide-treated arterial segments from leptin-treated or obese rats was markedly enhanced in comparison to the control group (Fig. 8B, Table 6).

**Table 6:** GYY4137-induced relaxation of phenylephrine-preconstricted mesenteric artery rings in control, leptin-treated and obese rats.

	Apamin+TRAM-34		Glibenclamide	
Group	R_max_ (%)	EC_50_ (µM)	R_max_ (%)	EC_50_ (µM)
Control	47.7±7.4	140.1±12.6	29.4±3.8	79.9±17.4
Leptin-treated	49.8±8.1	154.2±14.3	46.2±6.4***	88.2±15.3
Obese	49.4±7.9	149.2±13.7	48.1±5.9***	80.6±14.2

Concentration-dependent effect of GYY4137 was examined in the presence of apamin and TRAM-34 (K_ATP_ channel-dependent relaxation) or glibenclamide (SK_Ca_ and IK_Ca_ channel-dependent relaxation).

*** p<0.001 vs. control group (ANOVA and Tukey test).

## Discussion

The in vitro activity of the both non-pegylated and pegylated superactive rat leptin antagonists (D23L/L39A/D40A/F41A) was similar to that of similar mutants of mouse, human [28] and ovine [22] indicating that the similarity of the effect of the D23L mutation in different species is likely related to the fact that the sequence of amino acids 6–28 (VQDDTKTLIKTIVTRINDISHTQ), making up the main part of the first α-helix, is identical in human, mouse, rat, ovine, bovine and pig leptins. Though the 3D structure of leptin:leptin receptor complex has not yet been elucidated and an accurate structural interpretation of our findings is impossible, we suggested that D23 which is located at the C-terminal end of helix is part of the leptin binding site II [34]. Its replacement by a non-negatively charged amino acid probably abolishes some as yet unidentified repulsive effect and therefore increases the interaction with leptin receptor.

The weight gain effect of PEG-SRLA during the one week treatment of control rats was highly significant, similarly to the effect seen in various mice strains [28], [35] indicating similar reactivity at least in rodents. The effect in obese rats was lesser confirming our similar unpublished observations in mice fed high fat diet (A. Gertler, unpublished data). Notably, in 4 out of 6 PEG-SRLA-injected rats kept on high energy diet the weight gain was similar to that in the PEG-SRLA-injected control rats indicating individual variability in rats fed high fat diet.

The results of this study confirm our previous findings [17], [18] that: (1) chronic hyperleptinemia either induced in lean rats by administration of exogenous leptin or resulting from diet-induced obesity impairs acute NO-mediated vasodilating effect of leptin, (2) EDHF-dependent effect of leptin is up-regulated under these circumstances and compensates for NO deficiency. In addition, we obtained several new results. Most importantly, superactive leptin receptor antagonist restored leptin-induced NO signaling in obese rats although it did not reduce but rather tended to further increase body weight, which suggests that NO deficiency results from hyperleptinemia rather than from changes of other adipokines or metabolic abnormalities associated with obesity. Previously, leptin antagonists have been demonstrated to be beneficial in some mice models of disorders associated with metabolic syndrome or hyperleptinemia such as non-alcoholic fatty liver disease [36] or chronic kidney disease-induced lean body mass loss (Gertler A et al, unpublished observation). The results presented here suggest that leptin antagonists may improve NO-dependent vascular function in hyperleptinemic states. It remains to be established if leptin antagonists positively affect NO-mediated vasorelaxation in response to agonists other than leptin such as acetylcholine.

Second, we demonstrated that NO and COX-independent (i.e. EDHF-mediated) portion of leptin-induced vasorelaxation is mediated, at least in part, by H_2_S. Initial studies about vascular effect of H_2_S suggested that this gasotransmitter is produced only in smooth muscle cells and induces vasorelaxation by activating K_ATP_ channels in these cells. However, more recently it has been demonstrated that CSE is expressed in endothelial cells of peripheral arteries and that H_2_S mediates acetylcholine-induced EDHF-dependent relaxation of mouse and rat mesenteric arteries [37], [38], rat cerebral arteries [39] and mouse ductus arteriosus [40]. Indeed, acetylcholine stimulates CSE-dependent H_2_S production in endothelial cells, and H_2_S hyperpolarizes these cells by activating SK_Ca_ and IK_Ca_ channels [37]. Hyperpolarization of endothelial cells is then transmitted to smooth muscle cells through the myoendothelial gap junctions. In addition, opening of endothelial K^+^ channels may hyperpolarize smooth muscle cells by two other mechanisms: (i) activation of Na^+^,K^+^-ATPase, (ii) activation of K^+^ influx to smooth muscle cells through the inwardly-rectifying K^+^ channels (K_ir_); both effect result from local increase in extracellular K^+^ [31]. Our findings are consistent with this mechanism since the H_2_S donor, GYY4137, relaxed mesenteric arteries with either intact or removed endothelium and in endothelium-intact segments its effect was partially inhibited by apamin/TRAM-34 or glibenclamide and completely blocked by all three inhibitors. In addition, EDHF-dependent portion of leptin-induced vasorelaxation was markedly inhibited by either BSS or PAG suggesting the involvement of H_2_S. To the best of our knowledge this study is the first in which the involvement of H_2_S in vascular effect of leptin was demonstrated. The mechanism downstream from SK_Ca_ and IK_Ca_ channels was not examined by us. Thus, it is unclear if myoendothelial gap junctions, sodium pump or K_ir_ channels are involved in leptin-induced vasorelaxation. Our labs are currently investigating these possibilities.

Some studies suggest that, in contrast to large arteries, in smaller vessels the effect of H_2_S on smooth muscle cells is mediated by KCNQ rather than K_ATP_ channels. For example, Schleifenbaum et al. [41] have reported that anticontractile effect of NaHS on mouse mesenteric artery is prevented by the KCNQ inhibitor, XE991 but not by glibenclamide. In the present study the effect of GYY4137 on endothelium-denuded segments was completely abolished and on endothelium-intact segments was markedly attenuated by glibenclamide suggesting that the effect of H_2_S on smooth muscle cells was mediated, at least in part, by K_ATP_ channels. We did not address the possible involvement of KCNQ channels. It should be noted that in rat aortic rings vasodilating effect of NaHS was blocked by both XE991 and glibenclamide [42]. It is possible that both types of K^+^ channels are involved in the effect of H_2_S or that inhibitors used exhibit some non-specific activities toward K_ATP_ and KCNQ channels. In addition, our study was performed in the rat whereas Schleifenbaum et al. [41] examined mouse mesenteric arteries. Finally, different vasoconstrictors (α-adrenergic agonist vs. serotonin) were used in both studies. It should be noted that in the early study [43], relaxing effect of H_2_S in the ex vivo perfused rat mesenteric vasculature was partially attenuated by either endothelial denudation or by apamin and charybdotoxin (the other inhibitor of IK_Ca_) and partially reduced by glibenclamide, which is consistent with our results obtained by the measurement of isometric vascular tension.

In our previous study [44] we suggested that the EDHF-dependent vascular effect of leptin was mediated by cytochrome P450 (CYP)-dependent arachidonate derivatives such as EET because it was blocked by CYP inhibitor, sulfaphenazole. However, in the present study the effect of leptin was not affected by the other CYP inhibitor, SKF 525A. In that previous study [44] we did not examine isolated vessels but only blood pressure changes in rats injected with leptin and/or respective inhibitors, and it is possible that EETs might be involved in the effect of leptin in some other vascular beds. In addition, nonspecific effects of sulfaphenazole after its in vivo administration could not be excluded. Finally, EETs stimulate endothelial cell transient receptor potential vanilloid-4 (TRPV4) calcium channels which mediate Ca^2+^ influx in response to shear stress; this is the upstream mechanism contributing to activation of endothelial SK_Ca_ and IK_Ca_ channels [45]. Thus, we cannot completely exclude the possibility that, in addition to H_2_S, EETs are required for leptin-induced SK_Ca_ and IK_Ca_ stimulation.

The third important finding of this study is that in experimental obesity not associated with insulin resistance or hyperlipidemia the leptin-induced EDHF/H_2_S pathway is up-regulated and may compensate for NO deficiency. This effect is also accounted for by hyperleptinemia because was abolished by PEG-SRLA. The mechanism of EDHF up-regulation is unclear. Basal vascular CSE activity toward either cystathionine or cysteine were not different between groups, although we cannot exclude the possibility that in response to acute leptin treatment the enzyme was activated to the greater extent in leptin-treated and obese than in the control group. Since H_2_S signaling in tissues is markedly dependent on mitochondrial oxidation of the gasotransmitter, we also examined the effect of leptin in the presence of stigmatellin. Stigmatellin slightly enhanced the EDHF-dependent effect of leptin. In particular, stigmatellin reduced EC_50_ which would be consistent with accumulation of more H_2_S at lower leptin concentrations. However, the difference in leptin-induced vasorelaxation between control, leptin treated and obese groups persisted in the presence of stigmatellin, suggesting that impaired mitochondrial H_2_S oxidation in the two latter groups was not responsible for the enhancement of leptin-induced EDHF in our current study. It is well known that EDHF is inhibited by NO under baseline conditions and is often up-regulated in states of NO deficiency [31]. In addition, NO has been demonstrated to inhibit vascular CSE [37]. However, it is unlikely that up-regulation of EDHF/H_2_S in leptin-treated or obese groups resulted solely from NO deficiency because EDHF-dependent vasorelaxation was examined in the presence of NOS inhibitor in all preparations. The most likely explanation is that the sensitivity of endothelium to H_2_S was enhanced in leptin-treated and obese rats because GYY4137, which generates very low levels of H_2_S [26], [27], but not “decomposed” GYY4137, produced more marked relaxing effect in these groups. Previously it has been demonstrated that although EDHF-mediated effect of acetylcholine on the 4^rd^ order mesenteric arteries was impaired in obese rats fed the high-fat diet, IK_Ca_ activator, 1-EBIO, produced greater vasorelaxation in obese than in lean animals which was associated with greater expression of IK_Ca_ protein in the vessel wall [46]. An alternative explanation may be that the sensitivity to H_2_S was specifically increased in obesity. Indeed, plasma levels of H_2_S are known to be decreased in animal models of obesity and in obese humans where plasma H_2_S levels negatively correlated with physical indices of obesity, systemic blood pressure and microcirculatory function in vivo [47], [48]. Chronic deficiency of endogenous H_2_S could make blood vessels more sensitive to exogenous H_2_S donors or to the acute increase in endogenous H_2_S induced by vasodilators such as leptin. It is thought that many of the biological effects of H_2_S are mediated, at least in part, by the sulfhydration of cysteine –SH groups, and oxidized sulfenic (-SOH) groups are highly susceptible for sulfhydration in vitro [49]. As hyperleptinemia and obesity are associated with oxidative stress [50], conversion of thiol to sulfenic groups could render them more sensitive to sulfhydration.

In conclusion, we demonstrated that leptin relaxes peripheral arteries in both NO- and EDHF-dependent manner and the latter mechanism is mediated by H_2_S. Short-term obesity associated with hyperleptinemia but normal insulin sensitivity, glycemia and lipid profile impairs NO-dependent and augments EDHF-dependent effect of leptin. Both decrease in NO and increase in EDHF are corrected by leptin antagonist suggesting that they are accounted for by chronic hyperleptinemia but not by other consequences of obesity. Increase in leptin-induced EDHF/H_2_S pathway is mainly associated with the increased sensitivity of endothelium to H_2_S.
